# A 45-Year-Old Woman With Hereditary Hemorrhagic Telangiectasia and Persistent Exertional Dyspnea and Peripheral Edema

**DOI:** 10.1016/j.chest.2025.08.004

**Published:** 2026-01-08

**Authors:** Helen Triantafyllidi, Dionysia Birmpa, Anastasia Fambri, Dimitrios Benas, David Montani

**Affiliations:** a 2nd Department of Cardiology, National and Kapodistrian University of Athens, Medical School, ATTIKON Hospital, Athens, Greece; b Department of Respiratory and Intensive Care Medicine, Pulmonary Hypertension National Referral Centre, Université Paris-Saclay, AP-HP, INSERM UMR_S 999, Hôpital Bicêtre, Le Kremlin-Bicêtre, France

## Abstract

We report the case of a 45-year-old woman who was referred to our Cardiology Department because of persistent exertional dyspnea and peripheral edema. She had an established clinical diagnosis of hereditary hemorrhagic telangiectasia with multiple gastrointestinal telangiectasias that had been submitted to repeat embolization in the past and arteriovenous malformations in the liver and lungs. Complete blood count was diagnostic for severe anemia (hemoglobin 5-6 g/dL). Since the hereditary hemorrhagic telangiectasia diagnosis 3 years prior, the patient informed us that she has undergone 27 blood transfusions and multiple embolizations to manage gastrointestinal telangiectasias. Given her severe anemia that was caused by gastrointestinal telangiectasia, treatment with bevacizumab was initiated. Bevacizumab was administered over 8 cycles (initially biweekly for 4 doses, followed by monthly administration).

## Physical Examination Findings

At the time of admission, the patient was in New York Heart Association functional class III, and her vital signs included BP of 100/70 mm Hg, heart rate of 100 beats/min, and a respiratory rate of 16 breaths/min. Although she had an oxygen saturation of 93% on room air, desaturation to 88% was noted with exertion (walking for just a few meters). Her physical examination revealed a near normal auscultation of the lungs, severe bilateral peripheral edema in the lower limbs, and bilateral jugular vein distention. No signs of skin or mucosal telangiectasias were found.

**Figure 1:**
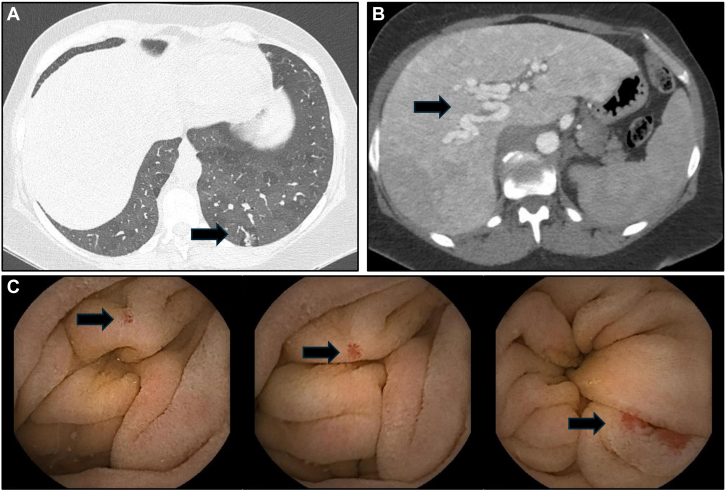
A-C, Arrows indicate (A) arteriovenous malformation in the chest CT scan in left lower lobe; (B) abdomen CT scan with multiple hepatic arteriovenous malformations: the largest 3.5 cm, and (C) multiple gastrointestinal telangiectasias during endoscopy.

## Diagnostic Studies

At the time of the first admission of the patient, transthoracic echocardiography (TTE) revealed right ventricular and atrial dilatation, a severe reduction of right ventricle systolic function, and an elevated right ventricle systolic pressure (70 to 75 mm Hg).

CT pulmonary angiography revealed a diffuse mosaic attenuation pattern and a small arteriovenous malformation (AVM) in the right lower lobe, with no evidence of chronic thromboembolic disease. Abdominal CT scan showed a large hepatic AVM (Fig 1).

Right heart catheterization (RHC) revealed mean pulmonary arterial pressure of 48 mm Hg, right atrial pressure of 19 mm Hg, pulmonary artery wedge pressure of 16 mm Hg, cardiac output (CO) of 9.4 L/min, cardiac index of 4.7 L/min/m^2^, and pulmonary vascular resistance of 3 Wood units.

Genetic testing identified a pathogenic variant in the *activin receptor like-protein 1* (*ACVRL1*) gene, which is associated primarily with autosomal dominant inheritance in hereditary hemorrhagic telangiectasia (HHT) and, less frequently, with pulmonary arterial hypertension (PAH), whether accompanied by HHT signs or not.

*What is the diagnosis?*

*Diagnosis:* Findings from RHC in this patient with HHT revealed combined before and after capillary pulmonary hypertension (PH) associated with high cardiac output according to the 2022 European Society of Cardiology/European Respiratory Society guidelines for the diagnosis and treatment of PH.

## Discussion

HHT, an autosomal dominant disorder that disrupts normal vascular morphogenesis, leads to a spectrum of vascular anomalies and severe anemia. HHT encompasses a spectrum of vascular anomalies that range from small, fragile telangiectasias prone to bleeding to large AVMs that can occur in various organs, which include the liver, lungs, and brain. HHT type 1 is associated with a higher prevalence of pulmonary and cerebral AVMs, mucocutaneous telangiectasia, and epistaxis; HHT type 2 leads to a higher prevalence of hepatic AVMs. AVMs carry a risk of rupture and hemorrhage, thereby increasing the morbidity and mortality rates that are associated with the condition. Approximately 90% of HHT is caused by heterozygous mutations in the *endoglin* gene (type 1 HHT) or *ACVRL1* gene (type 2 HHT). These genes encode transmembrane receptors that, together with other receptors, such as bone morphogenetic protein receptor II, activate transcriptional responses in endothelial cells.

PH may occur as a complication of HHT in a substantial percentage of patients with HHT. PH may occur as a complication of HHT in 14% to 45% of patients with HHT in studies that are based on TTE, although the true prevalence is significantly lower in RHC-based studies. PAH in HHT may arise from multiple mechanisms, such as heart failure and PH secondary to high CO caused by multiple AVMs or, less frequently, heritable PAH caused by pulmonary arterial remodeling. At least, 3 mechanisms contribute to PH in this patient: precapillary PAH, high CO, and postcapillary PH that was the result of the high CO.

Bevacizumab, a recombinant humanized monoclonal IgG1 antibody, targets vascular endothelial growth factor (VEGF), a cytokine elevated in HHT, and may restore anemia. Indeed, IV bevacizumab can improve severe anemia caused by epistaxis and/or refractory gastrointestinal bleeding. The proposed mechanism involves inhibition of the VEGF-mediated neoangiogenesis and a reduction in capillary fragility. VEGF is abundant in the lung and plays a critical role in maintaining pulmonary vascular structure and function.

Plasma VEGF levels are elevated in patients with severe PAH and both VEGF and its receptor 2 (VEGFR 2) are expressed strongly in the complex vascular lesions of PAH lungs. However, whether VEGF plays a mechanistic role in the development of PAH remains unclear. Notably, animal models have shown that treatment with VEGF receptor blockers, particularly under hypoxic conditions, can induce severe angio-obliterative PAH, which highlights the dual angiogenic and antiangiogenic potential of VEGF, depending on ligand-receptor interactions and signaling context.

Several drugs have been associated to the onset or worsening of PAH. Bevacizumab may have a contributing role in PAH development because of angiogenesis impairment, dysregulation of the immune system, and increasing proliferation/survival of pulmonary arterial smooth muscle. In humans who receive bevacizumab for colorectal cancer, hyperproliferative endothelial cells may occlude the pulmonary vascular lumen, which underscores the importance of intact VEGF signaling for the maintenance of pulmonary vascular integrity. Interestingly, contrary evidence from hypoxia-induced PH models in rats suggests bevacizumab might mitigate PH under certain conditions. Bevacizumab has been shown to improve or partially normalize high CO in some patients with HHT. However, its effect on CO is not consistently observed. Improvement in CO tends to occur when bevacizumab treatment is initiated at a stage of milder disease, characterized by asymptomatic elevated CO without heart chamber enlargement. Recently, at the 7th World Symposium on Pulmonary Hypertension, bevacizumab was included in the list of drugs and toxins possibly associated with PAH; several cases have been reported.

### Clinical Course

Treatment by bevacizumab achieved hemoglobin stability (10 to 11 g/dL) for approximately 10 months; however, serial TTE revealed a progressive increase in right ventricle systolic pressure (75 to 80 mm Hg) and a decline in CO. The patient experienced recurrent hospitalizations for right-sided heart failure, presenting with pleural effusion, ascites, and peripheral edema, that required high doses of IV diuretics and inotropes. She succumbed to refractory right-sided heart failure and sepsis 2 years after her initial admission and 1 year after the last dose of bevacizumab. Bevacizumab may be considered as an effective treatment modality of refractory bleeding in patients with HHT. However, in case of PH co-existing with HHT, treatment by bevacizumab probably deteriorates PH by inducing pulmonary vascular remodeling.

## Clinical Pearls

1. *HHT, an autosomal dominant disorder that disrupts normal vascular morphogenesis, leads to a spectrum of vascular anomalies and severe anemia.*
2. *PH may occur as a complication of HHT in a substantial percentage of patients with HHT.*
3. *Bevacizumab, a recombinant humanized monoclonal IgG1 antibody, targets VEGF, a cytokine that is elevated in HHT and may restore anemia.*
4. *Bevacizumab may have a contributing role in PAH development caused by angiogenesis impairment, dysregulation of the immune system, and increasing proliferation/survival of pulmonary arterial smooth muscle.*
5. *In case of PH co-existing with HHT, treatment by bevacizumab probably deteriorates PH by inducing pulmonary vascular remodeling.*

## Financial/Nonfinancial Disclosures

The authors have reported to *CHEST* the following: H. T. received payments/honoraria as a speaker from Menarini, Merck and consulting fees from Servier, Menarini, Galenica. D. M. received payments/honoraria as a speaker from Bayer, Janssen, Boerhinger, Chiesi, GSK, Ferrer, Merck MSD, grants from Acceleron, Janseen, Merck MSD and consulting fees from Acceleron, Janseen, Merck MSD, Ferrer. None declared (D. B., A. F., D. B.).
